# Effect of Acute Hypoxia on Post-Exercise Parasympathetic Reactivation in Healthy Men

**DOI:** 10.3389/fphys.2012.00289

**Published:** 2012-07-25

**Authors:** Hani Al Haddad, Alberto Mendez-Villanueva, Pitre C. Bourdon, Martin Buchheit

**Affiliations:** ^1^Physiology Unit, Sport Science Department, Aspire Academy for Sports ExcellenceDoha, Qatar

**Keywords:** hypoxia, vagal-related indices, autonomic activity, post-exercise recovery

## Abstract

In this study we assessed the effect of acute hypoxia on post-exercise parasympathetic reactivation inferred from heart rate (HR) recovery (HRR) and HR variability (HRV) indices. Ten healthy males participated in this study. Following 10 min of seated rest, participants performed 5 min of submaximal running at the speed associated with the first ventilatory threshold (Sub) followed by a 20-s all-out supramaximal sprint (Supra). Both Sub and Supra runs were immediately followed by 15 min of seated passive recovery. The resting and exercise sequence were performed in both normoxia (N) and normobaric hypoxia (H; FiO_2_ = 15.4%). HRR indices (e.g., heart beats recovered in the first minute after exercise cessation, HRR_60s_) and vagal-related HRV indices [i.e., natural logarithm of the square root of the mean of the sum of the squared differences between adjacent normal *R*–*R* intervals (Ln rMSSD)] were calculated for both conditions. Difference in the changes between N and H for all HR-derived indices were also calculated for both Sub and Supra. HRR_60s_ was greater in N compared with H following Sub only (60 ± 14 vs. 52 ± 19 beats min^−1^, *P* = 0.016). Ln rMSSD was greater in N compared with H (post Sub: 3.60 ± 0.45 vs. 3.28 ± 0.44 ms in N and H, respectively, and post Supra: 2.66 ± 0.54 vs. 2.65 ± 0.63 ms, main condition effect *P* = 0.02). When comparing the difference in the changes, hypoxia decreased HRR_60s_ (−14.3% ± 17.2 vs. 5.2% ± 19.3; following Sub and Supra, respectively; *P* = 0.03) and Ln rMSSD (−8.6% ± 7.0 vs. 2.0% ± 13.3, following Sub and Supra, respectively; *P* = 0.08, Cohen’s effect size = 0.62) more following Sub than Supra. While hypoxia may delay parasympathetic reactivation following submaximal exercise, its effect is not apparent following supramaximal exercise. This may suggest that the effect of blood O_2_ partial pressure on parasympathetic reactivation is limited under heightened sympathetic activation.

## Introduction

Exercising in altitude has grown in popularity in recent years. For example, the number of registered participants in the international Ultra-Trail of the Mont Blanc has increased from 700 in 2003 up to 10,000 in 2012 (http://www.ultratrailmb.com/documents/Newsletters/Letters/nl2_fevrier2012_EN.html). Hypoxia, which is characterized by a decrease in the inspired O_2_ pressure due to lower ambient air pressure is a strong environmental stressor that elicits cardiovascular compensation and adaptation [e.g., increasing resting heart rate (HR) and respiratory rate, and reducing systemic blood pressure; Rowell et al., 1989; Halliwill et al., 2003]. Importantly, hypoxia is thought to stimulate peripheral chemoreceptors and to increase local vasodilatation (i.e., decrease blood pressure), which leads to an increase in sympathetic/decrease in parasympathetic activity (Somers et al., 1989b; Kara et al., 2003), affecting, in turn, HR dynamics. Since an increased sympathetic activity and/or a delayed parasympathetic reactivation following exercise has been associated with an increased risk of sudden cardiac death (Billman, 2002), exercising in a hypoxic environment is therefore likely to increase the prognosis of developing cardiac arrhythmia (Clark et al., 1963). While the acute effects of hypoxia on cardiac autonomic nervous system function have been well investigated at rest and during exercise (Somers et al., 1989a; Yamamoto et al., 1996, Perini and Veicsteinas, 2003; Buchheit et al., 2004, Iwasaki et al., 2006), the effect of hypoxia on parasympathetic reactivation following exercise has, to our knowledge, never been documented.

Beside environmental conditions, exercise intensity itself is an important determinant of post-exercise parasympathetic reactivation. Compared with submaximal exercise, maximal or supramaximal exercises are associated with slower parasympathetic reactivation (Buchheit et al., 2007a; Seiler et al., 2007). For example, a vagal-related heart rate variability (HRV) index, i.e., the rMSSD (square root of the mean of the sum of the squares of differences between adjacent normal *R*–*R* intervals) was ∼40% lower following high-intensity compared with submaximal running (Buchheit et al., 2007a). In addition to central command (Kaufman, 2010), metaboreflex and chemoreflex activation due to exercise-induced blood metabolite accumulation during the post-exercise recovery period (e.g., lactate, plasma epinephrine, inorganic phosphate (Vissing, 2000; Kaufman and Hayes, 2002) are likely responsible for the delayed parasympathetic reactivation following intense exercise (Buchheit et al., 2007a). Therefore, it can be speculated that a combination of hypoxia and high-intensity exercise (both stimulating sympathetic activity) might be associated with a very limited parasympathetic reactivation. Nevertheless, this still needs to be examined.

Therefore, to better understand the respective impact that hypoxia and exercise intensity have on post-exercise autonomic activity, we examined the time course of parasympathetic reactivation following submaximal and supramaximal exercise performed both in normoxic and hypoxic conditions. We first hypothesized that, regardless of exercise intensity, the additional activation of chemoreceptors via decreased ambient air O_2_ pressure would delay post-exercise parasympathetic reactivation. We also expected that the supramaximal exercise performed under hypoxic conditions would result in the slowest parasympathetic reactivation.

## Materials and Methods

### Participants

Ten healthy males (32.7 ± 4.0 years; 1.81 ± 0.06 m; 81.0 ± 5.1 kg; training 4–8 h per week) volunteered for the study. They underwent a medical pre-screening and provided an informed written consent prior the participation in this study. All participants were familiar with the protocol and were not taking prescribed medications and presented with normal blood pressure levels and electrocardiographic patterns. They were instructed to avoid any physical exertion during the 24-h proceeding each test session. The study conformed to ethical guidelines outlined in the Declaration of Helsinki and was approved by the local institution’s human research ethics committee.

### Study overview

Participants completed all tests within a 3-week period. All tests were performed at Aspire academy (Doha, Qatar) laboratory where ambient temperature ranged from 20 to 23°C. Participants first performed an incremental running test to determine their maximal O_2_ uptake ($\dot{V}{\text{O}}_{2}$ max) and associated velocity (v$\dot{V}{\text{O}}_{2}$ max), as well as the first ventilatory threshold (*V*_Th1_) and associated velocity (v*V*_Th1_). During two consecutive weeks, participants performed an exercise sequence in either normoxia (N) or hypoxia (H). The normobaric H condition was set to simulate an altitude of 2400 m (FiO_2_ = 15.4%) and was created using a CAT system (Colorado altitude training, Louisville, CO, USA). This system duplicates the lower O_2_ pressure found at high altitude by lowering the percentage of O_2_ in the air. Blood O_2_ saturation (SpO_2_) was continuously monitored using wrist worn oxymeter (Nonin 3100, Nonin Medical Inc., Plymouth, MN, USA). SpO_2_ values averaged over a 3-s period were recorded at 5 and 10 min during the rest period and at 3, 5, 10, and 15 min during the post-exercise recovery periods. HR recordings (s810 HR monitor, Polar Electro, Kemple, Finland) were carried out during resting periods and throughout the entire exercise sequence. All tests were conducted on the same day of the week and at the same time of day. All participants were asked to consume their last meal at least 3 h before each test session. Although respiratory rate is often controlled for HRV studies, we chose not to control respiratory rate in our participants because we did not want to interfere with the natural return of HR to baseline levels. Moreover, vagal-related HRV indices during spontaneous or controlled breathing differ little (Bloomfield et al., 2001).

### Incremental running test

The incremental running test was performed on a motorized treadmill (Woodway PPS Med, Woodway, Waukesha, WI, USA) under normoxic conditions (FiO_2_ = 20.9%). The test began at an initial speed of 8 km h^−1^ (+1% slope), which was increased by 1 km h^−1^ every minute until exhaustion. During the test, respiratory gas exchange was measured breath-by-breath using an Oxycon metabolic system (Carefusion GmbH, Hoechberg, Germany). Prior to each test the O_2_ and CO_2_ analyzers were calibrated as recommended by the manufacturer. Minute ventilation (VE), O_2_ uptake ($\dot{V}{\text{O}}_{2}$), and carbon dioxide production($\dot{V}{\text{CO}}_{2}$) were computed. Cardiorespiratory values were averaged over 30 s periods during the incremental running test. $\dot{V}{\text{O}}_{2}$ max was considered as the highest $\dot{V}{\text{O}}_{2}$ value attained in a 30-s period. In addition, tests were considered maximal when: (1) participants’ maximal HR (HR_max_) was near their age-predicted maximal HR (i.e., 220-age ± 10 beats min^−1^), (2) blood lactate concentration was higher than 8 mmol l^−1^, and (3) respiratory exchange ratio (RER) was greater than 1.10. VE/$\dot{V}{\text{O}}_{2}$ and VE/$\dot{V}{\text{CO}}_{2}$ were plotted vs. running velocity. *V*_Th1_ corresponded to the first deflection point of increase in the VE/$\dot{V}{\text{O}}_{2}$ curve with VE/$\dot{V}{\text{CO}}_{2}$ slope remained constant (Reinhard et al., 1979). Detection of *V*_Th1_ was made by visual inspection of the graphs by two experienced exercise physiologists.

### Exercise sequence

Following 10 min of seated rest, participants performed a 5-min submaximal run (Sub) and a 20-s all-out supramaximal sprint (Supra). Each Sub and Supra run was immediately followed by 15 min of passive seated recovery. The exercise sequence, including the resting period, was performed both in H and N conditions. HR measurements in H started as soon as the participant assumed the seated position in the hypoxic chamber (i.e., within 30 s after entering the hypoxic chamber) to perform the resting sequence where HR-derived indices were calculated from the last 5 min of the 10-min resting period.

### Submaximal and supramaximal running exercises

**Figure 1:**
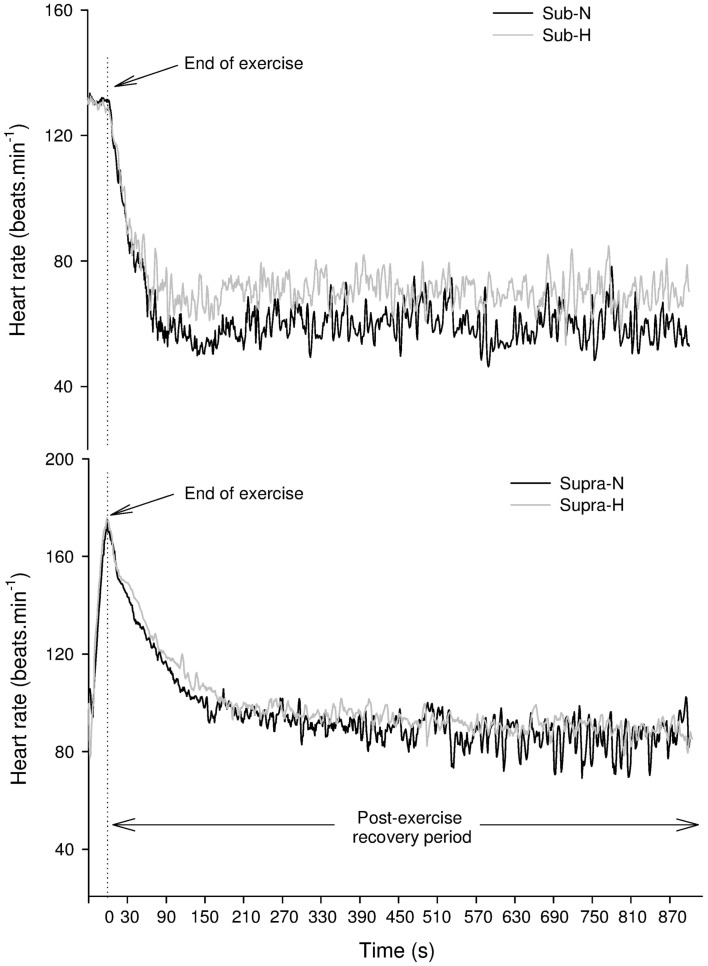
**Changes in heart rate in a representative subject following submaximal (Sub – upper panel) and supramaximal (Supra – lower panel) exercise (lower panel) in normoxic (N) and hypoxic (H) conditions**.

Participants performed 5 min of submaximal running at v*V*_Th1_ on a motorized treadmill (Woodway PPS Med, Woodway, Waukesha, WI, USA). This intensity was chosen to ensure rapid return of HR to baseline levels following exercise (Buchheit et al., 2009), as required for appropriate short-term post-exercise HRV analysis (i.e., steady-state signal; Figure 1). Importantly, in order to assume similar exercise-induced sympathetic activation during Sub, running velocity of Sub during H was adjusted to match the participant’s HR attained during Sub in N (i.e., calculated over the last 30 s of exercise).

Prior to the study, all participants were familiarized with sprinting on the non-motorized treadmill. Prior to the Supra test (i.e., 20 s all-out sprint), participants performed a 2-min warm-up at v*V*_Th1_ on the motorized treadmill (Woodway PPS Med, Woodway, Waukesha, WI, USA). Participants then performed two maximal (2–3 s) sprints. During the warm-up and the 20-s all-out sprints, a harness was placed around participant’s waist, which was attached to a vertical bar at the rear of the treadmill. This ensured that the subjects maintained a constant position on the treadmill. The harness height was adjusted to avoid interference while running. A 1-min break separated the warm-up from the 20-s all-out sprint. No additional resistance was applied during the sprint and participants were verbally encouraged to perform maximally. Peak exercise HR during the sprint was considered as the highest value attained over a 5-s period. Maximal running speed (km h^−1^) reached during the sprint was obtained from the certified treadmill software. Blood acidosis/lactate accumulation are likely to influence post-exercise HRR and HRV indices (Buchheit et al., 2007a), hence the 20-s all-out running exercise was chosen since it induces similar lactate level in hypoxic and normoxic conditions (Ogura et al., 2006).

### Data measurement and analyses

#### Heart rate measurements

HR data were downloaded on a computer using compatible Polar software (Polar Precision Performance SW 5.20, Polar Electro, Kempele, Finland). All irregular heart beats were automatically identified and replaced with interpolated adjacent *R*–*R* interval values with the Polar Software (Nunan et al., 2009). The default setting of a minimum beat protection zone of 6 beats min^−1^ was used (Nunan et al., 2009).

#### HR-derived indices assessment

At rest HRV indices were calculated from the last stationary 5 min of the 10-min resting sequence. After exercise, HRV indices were assessed from the last stationary 10 min of each 15-min period. Time domain indices were the mean *R*–*R* intervals (mRR) and the rMSSD. Spectral analysis was conducted to obtain the power spectral densities in the low (≥0.04–0.15; LF, mediated via both sympathetic and parasympathetic systems), and high frequencies (>0.15–0.40; HF, mediated via parasympathetic system under respiratory influence). The sympathovagal balance (LF/HF) was subsequently calculated. Peak HF (HF_peak_), which is associated with breathing frequency (Bernardi et al., 1990), was also computed. All HRV indices were calculated using Kubios HRV analysis software (Kubios HRV Analysis, Biosignal Analysis and Medical Imaging Group, Kuopio, Finland).

Heart rate recovery (HRR) was calculated by fitting the 15-min post-exercise beat-to-beat HR data into a first-order exponential decay curve (Buchheit et al., 2007b). A HR time constant (HRRτ) was then produced by modeling the resultant 15-min of HR data using an iterative technique (Sigmaplot 10; SPSS Science, Chicago, IL, USA) using the following equation: HR = HR_0_ + HRamp e^(−*T*/HRRτ)^, where HR_0_ is resting (final) HR, HRamp is maximal HR (HR_max_) − HR_0_, and *T* is time (s). HRR was also assessed by calculating the absolute difference between the final HR at exercise end and the HR recorded 60 s later (HRR_60s_; Buchheit et al., 2007b). However, since the (stabilized) end-recovery HR baseline likely determines the overall HR recovery amplitude, and, in turn, HRR_60s_, this latter index was also expressed as a percentage of the overall HR amplitude as follow: %HRR_60s_ = HRR_60s_ × 100/(HR at exercise end − end-recovery HR baseline measured over the last 10 min of the 15-min recovery period).

#### Time-varying vagal-related HRV index

A time-varying vagal-related index, the rMSSD_30s_, was calculated for each of the 30-s segments of recovery for the two 15 min recovery conditions (Goldberger et al., 2006). Data were median filtered in order to smooth out transient outliers in the HRV plots (HRV vs. time in recovery). The first and last values were not median filtered (Goldberger et al., 2006).

#### Blood sampling and analyses

Three minutes following the incremental running test, a fingertip blood sample (5 μl) was collected and the blood lactate concentration ([La]_b_) was determined (Lactate Pro, Arkay Inc, Kyoto, Japan; Pyne et al., 2000). The accuracy of the analyzer was checked before each test using standards. During the passive seated recovery periods immediately following each Sub and Supra run, arterialized capillary blood samples were collected from hyperemic fingertip. Blood samples were collected into 2.0 ml K2 EDTA vacuum tubes (Becton-Dickinson, Plymouth, UK) at 3, 5, 10, and 15 min during the post-exercise recovery phase. The analyses for blood gases (PaO_2_, PaCO_2_) and blood acidosis (pH) were performed immediately after collection using a ABL 77 blood gas analyzer (Radiometer Medical ApS, Copenhagen, Denmark).

#### Statistical analyses

The distribution of each variable was examined with the Kormoglov–Smirnov normality test. When data were skewed, natural logarithm transformation (Ln) was applied to HRV indices (e.g., Ln rMSSD) to obtain a normal distribution and allow parametric statistical comparisons. Differences in HRR, HRV indices blood variables, and time-varying vagal index between both conditions (i.e., H vs. N) for both exercise intensities were compared using a two-way ANOVA for repeated measures, with “intensity” (i.e., Sub and Supra) and “condition” (i.e., H and N) as factors. Further comparison of the time-varying Ln rMSSD_30s_ and blood variables (pH, PaCO_2_, PaO_2_, and SpO_2_) indices during post-exercise recoveries was conducted using a two-way ANOVA for repeated measures with “time” (i.e., measurements during rest and recovery) and “condition” (i.e., H and N) as factors. When a significant interaction was noted, a Bonferroni’s *post hoc* was conducted to further delineate the main effects and/or time effect during recovery and interactions between exercise intensities and recovery conditions. A paired *t*-test was performed to compare mean HR and running speed during submaximal and supramaximal exercises, and relative changes in the HR-derived indices in H compared with N for both submaximal and supramaximal exercises. Effect size (ES) was calculated for *P* ≤ 0.10. The magnitude of the ES was considered either small (>0.2), moderate (>0.5), or large (>0.8). If ES was at least moderate (>0.5), then the likelihood of a type II error was noted. Pearson’s coefficient of correlation was also calculated to assess the relationships between blood variables, HRV, and HRR indices. A multiple linear regression model with a stepwise backward elimination procedure was also used. Variables with *F*-value < 3.9 were removed from the model. The following criteria were adopted to interpret the magnitude of the correlation (*r*) between test measures: ≤0.1, trivial; >0.1–0.3, small; >0.3–0.5, moderate; >0.5–0.7, large; >0.7–0.9, very large; and >0.9–1.0, almost perfect. If the 90% confidence intervals overlapped positive and negative values, the magnitude were deemed unclear; otherwise that magnitude was deemed to be the observed magnitude (Hopkins et al., 2009). The *r*^2^ values derived from the multiple linear regression model were converted to *r* values in order to use the latter criteria to interpret the magnitude of the relationships. These analyses were performed using SigmaStat software (SigmaStat 3.11, Systat software inc., San Jose, Calif., USA). Significance level was set at *P* ≤ 0.05.

## Results

### Incremental running test

Mean values for $\dot{V}{\text{O}}_{2}$ max, RER, v$\dot{V}{\text{O}}_{2}$ max, HR_max_, [La]_b_ and v*V*_Th1_, were: 54.3 ± 6 ml min^−1^ kg^−1^, 1.13 ± 0.03, 17.2 ± 1.9 km h^−1^, 187 ± 9.1 beats min^−1^, 11.7 ± 1.6 mmol L^−1^, 10.3 ± 1.4 km h^−1^ (60.16 ± 3.25% of v$\dot{V}{\text{O}}_{2}$ max), respectively. The incremental running test was rated as maximal for all participants.

### Submaximal and supramaximal running exercise

During Sub, Mean HR (133 ± 9 vs. 135 ± 10 beats min^−1^ for N and H, respectively, *P* = 0.23) and running speed (10.3 ± 1.4 vs. 9.8 ± 1.3 km h^−1^ for N and H, respectively, *P* = 0.12; ES = 0.37) were not different between experimental conditions. For Supra, maximal HR (167 ± 9 vs. 168 ± 9 for N and H, respectively, *P* = 0.83) and the maximal running speed (28.8 ± 3.5 vs. 29.3 ± 3.6 km h^−1^ for N and H, respectively, *P* = 0.12) were also not different between conditions.

### SpO_2_, blood gases, and blood acidosis

#### Submaximal running exercise

**Figure 2:**
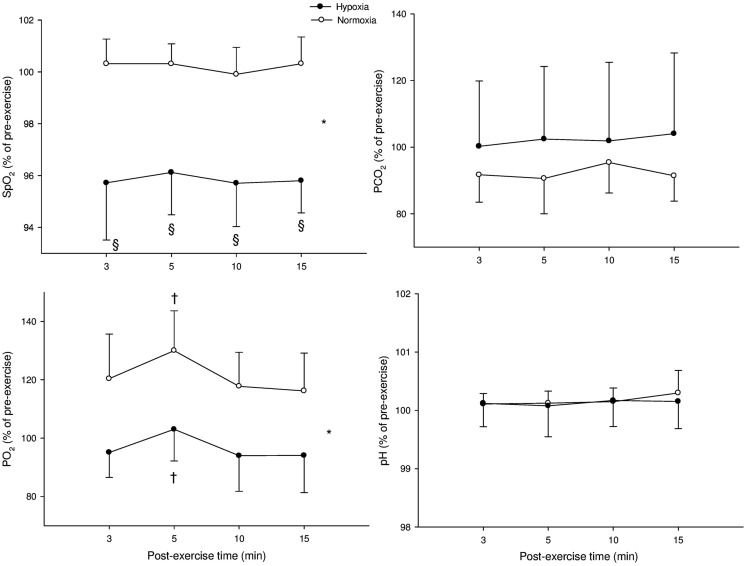
**Mean values (±SD) for arterial O_2_ saturation (SpO_2_), carbon dioxide partial blood pressure (PaCO_2_), O_2_ partial blood pressure (PaO_2_), and blood acidosis (pH) following submaximal exercise**. *: Significant general effect of condition; ^†^: within-condition difference vs. 3 min.

Figure 2 illustrates the time course of SpO_2_, blood gases, and blood acidosis in N and H. SpO_2_ was higher in N compared with H (*P* < 0.001) and an interaction was noted with SpO_2_ at 3, 5, 10, and 15 min being greater in N than in H (all *P* < 0.001). PaCO_2_ was not different in H when compared with N (*P* = 0.32). PaO_2_ was greater in N compared with H (*P* < 0.001) and a “time” effect was noted (PaO_2_ at 5 min was greater than PaO_2_ at 3 min in both H and N, *P* < 0.001). Blood pH was not different between N and H conditions (*P* = 0.88).

#### Supramaximal running exercise

**Figure 3:**
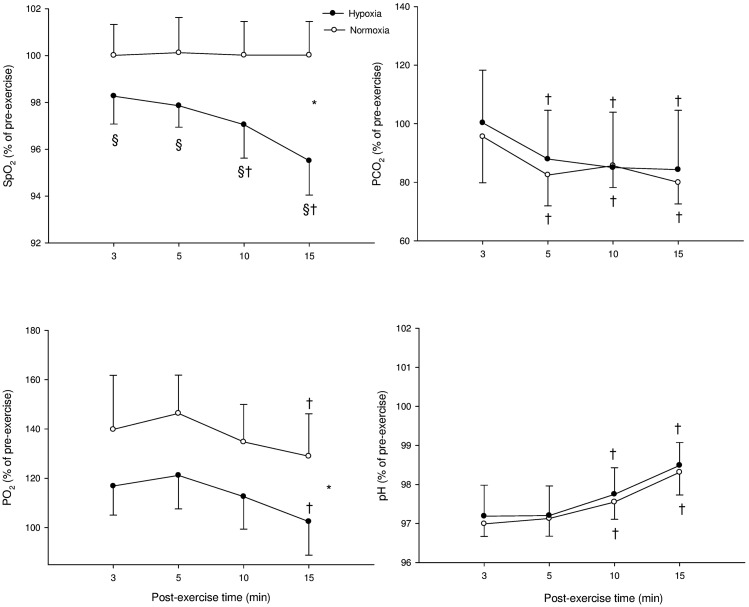
**Mean ± SD for arterial O_2_ saturation (SpO_2_), carbon dioxide partial blood pressure (PaCO_2_), O_2_ partial blood pressure (PaO_2_), and blood acidosis (pH) following supramaximal exercise**. *: Significant general effect of condition; ^†^: within-condition difference vs. 3 min; ^§^: significant difference vs. normoxia.

Figure 3 illustrates the time course of SpO_2_, blood gases, and blood acidosis in N and H conditions. SpO_2_ was greater in N compared with H at 3, 5, 10, and 15 min (*P* < 0.001). PaCO_2_ showed a “time” effect (PaCO_2_ at 5, 10, and 15 min were lower compared with PaCO_2_ at 3 min in both H and N; *P* < 0.001). PaO_2_ was greater in N compared with H (*P* < 0.001) and PaO_2_ was lower at 15 min compared with 3 min in both conditions (*P* < 0.001). Blood pH showed only a “time” effect in both conditions (pH at 10 and 15 min were greater than pH at 3 min in both H and N; *P* < 0.001).

When comparing the relative changes for hypoxia-induced changes following Sub and Supra, no difference was found for SpO_2_, blood gases, and pH (*P* = 0.56, 0.19, 0.73, 0.10 and for SpO_2_, PaCO_2_, PaO_2_, and pH, respectively).

### Heart rate measures

#### Resting HR-related indices

At rest, HR-derived indices were not significantly different in H compared with N: mRR (1125 ± 182 vs. 1164 ± 204 ms for H and N, respectively, *P* = 0.14), Ln rMSSD (4.0 ± 0.39 vs. 4.1 ± 0.21 for H and N, respectively, *P* = 0.32), LnHF (6.6 ± 0.71 vs. 6.6 ± 0.48 for H and N, respectively, *P* = 0.87), and LF/HF (2.9 ± 1.52 vs. 3.0 ± 1.58 for H and N, respectively, *P* = 0.86).

#### Post-exercise HRR indices

**Figure 4:**
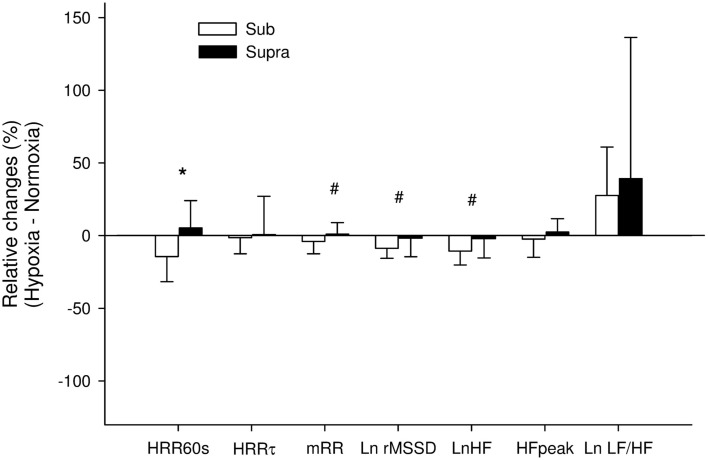
**Relative changes induced by hypoxia compared with normoxia following submaximal exercise (Sub) and Supra (supramaximal exercise) for absolute difference between the final heart rate (HR) at exercise end and the HR recorded 60 s later (HRR_60s_), time constant decay of HR recovery (HRRτ), mean R–R interval (mRR) and natural logarithm of the squares root of the mean of the sum of the squares of differences between adjacent normal *R*–*R* intervals (Ln rMSSD), natural logarithm of power spectral density in high frequency (LnHF), peak frequency of the HF power spectral density (HFpeak), and natural logarithm of the sympathovagal balance index (Ln LF/HF) calculated from the last 10 min of recovery**. *: Significant difference between Sub and Supra; #: difference between exercise intensity with effect size considered as moderate (>0.5).

Heart rate recovery indices for both exercise intensities during the two experimental conditions are reported in Table 1. Following Sub, HRR_60s_ was greater in N compared with H (*P* = 0.016). %HRR_60s_ was not significantly different between H and N following Sub (83% ± 19 vs. 76% ± 30 for N and H, respectively), and Supra (43% ± 10 vs. 45% ± 14 for N and H, respectively; main condition effect, *P* = 0.30). HRRτ was not significantly different between H and N (main condition effect, *P* = 0.91). The relative changes induced by H compared with N in HRR indices following both exercise intensities are presented in Figure 4. The hypoxia-induced reduction in HRR_60s_ was greater in Sub compared with Supra (−14.3% ± 17.2 vs. 5.2% ± 19.3; following Sub and Supra, respectively; *P* = 0.03). Similarly, hypoxia-induced a greater decrease in %HRR_60s_ for Sub compared with Supra (−7.0% ± 12.5 vs. −0.5% ± 7.1 following Sub and Supra, respectively, *P* = 0.10; ES = 0.60). In contrast, the effect of hypoxia on HRRτ was not significantly different between Sub and Supra (−1.4% ± 11.0 vs. 0.5% ± 27.2, following Sub and Supra, respectively; *P* = 0.42).

**Table 1:** **Heart rate recovery (HRR) and heart rate variability (HRV) indices following submaximal (Sub) and supramaximal (Supra) running exercise in normoxic and hypoxic environmental conditions**.

	Sub		Supra		Condition effect	Exercise intensity	Interaction
	Nomoxia	Hypoxia	Normoxia	Hypoxia			
**HRR INDICES**							
HRR_60s_ (beats min^−1^)	60 ± 14^#^	52 ± 19*^#^	36 ± 7	37 ± 10	0.12	<0.001 Sub > Supra	0.01
HRRτ (s)	24± 7	25± 9	91± 32	89± 47	0.91	<0.001 Sub < Supra	0.85
**HRV INDICES**							
mRR (ms)	980± 157	940± 155	726± 127	730± 130	0.24	<0.001 Sub > Supra	0.24
Ln rMSSD (ms)	3.6± 0.4	3.2± 0.4	2.6± 0.5	2.6± 0.6	0.02 N > H	0.002 Sub > Supra	0.36
LnHF (ms^2^)	5.7± 0.6	5.1± 0.7	3.8± 1.2	3.7± 1.1	0.03 N > H	0.003 Sub > Supra	0.17
HF_peak_ (Hz)	0.21 ± 0.04	0.20 ± 0.02	0.23 ± 0.04	0.24 ± 0.05	0.81	0.020 Sub < Supra	0.23
Ln LF/HF	1.7± 0.3	2.0± 0.4	2.2± 0.7	2.4± 0.6	0.01 N < H	0.030 Sub < Supra	0.37

Mean ± SD of absolute difference between the final heart rate at exercise end and the heart rate recorded 60 s later (HRR_60s_), time constant decay of heart rate recovery (HRRτ), mean *R*–*R* interval (mRR), and natural logarithm of the squared root of the mean of the sum of the squares of differences between adjacent normal *R*–*R* intervals (Ln rMSSD), natural logarithm of the power spectral density in high frequency (LnHF), peak frequency of the high frequency power spectral density (HF_peak_), and natural logarithm of the sympathovagal index (Ln LF/HF) calculated from the last 10 min of recovery. *, Significant within-exercise intensity difference; #, significant within-condition difference.

#### Post-exercise HRV indices

Visual examination of individual HR traces during the post-exercise recovery period confirmed the stableness of the analyzed period (Figure 1). Post-exercise HRV indices are reported in Table 1. All vagal-related HRV indices were greater following Sub compared with Supra, while Ln LF/HF was lower. Ln rMSSD and LnHF were greater in N compared with H (main condition effect, *P* = 0.02 and 0.03 for Ln rMSSD and LnHF, respectively), while Ln LF/HF was lower (main condition effect, *P* = 0.01). The relative changes induced by H compared with N for HRV indices following both exercise intensities are presented in Figure 4. The effect of H on mRR (−3.9% ± 8.4 vs. 0.8% ± 8.1, following Sub and Supra, respectively, *P* = 0.12; ES = 0.57), Ln rMSSD (−8.6% ± 7.0 vs. −2.0% ± 13.3, following Sub and Supra, respectively; *P* = 0.08, Cohen’s effect size = 0.62) and LnHF (−10.6% ± 9.5 vs. −2.4% ± 13.7, following Sub and Supra, respectively; *P* = 0.07; ES = 0.69) was greater after Sub than Supra.

#### Time-varying vagal-related HRV index

**Figure 5:**
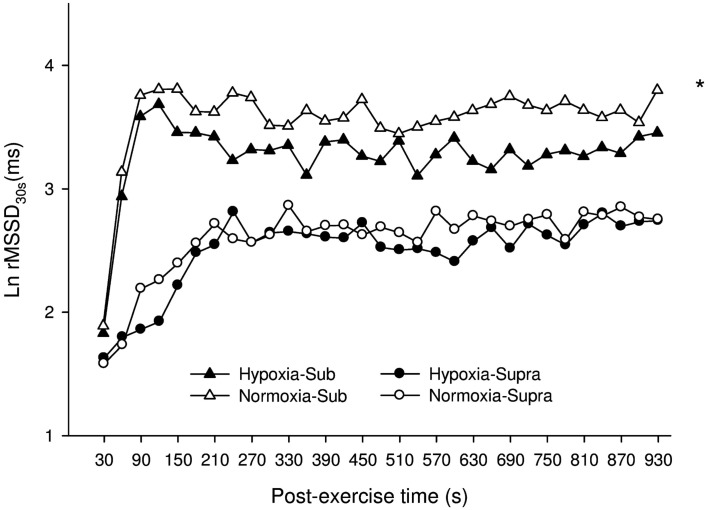
**Mean ± SD for the natural logarithm of the square root of the mean sum of the squared differences between adjacent normal *R*–*R* intervals measured on successive 30-s segments (Ln rMSSD_30s_) during the 15-min post-exercise recovery period, as calculated for participants in H (hypoxia, filled triangles and circles for Sub and Supra, respectively), or in N (normoxia, open triangles and circles for Sub and Supra, respectively)**. For figure clarity error bars were not presented. *: Significant general effect of condition. Analysis of the relative changes between H and N showed that H tended to have a greater effect after Sub than Supra (*P* = 0.07).

Figure 5 illustrates the time course of the time-varying Ln rMSSD_30s_ in N and H. For Sub, Ln rMSSD_30s_ was greater in N compared with H (main “condition” effect, *P* = 0.003). A “time” effect was also noted (*P* < 0.001). For Supra, no difference was found between conditions (main “condition” effect, *P* = 0.76), only a “time” effect was noted (*P* < 0.001). Analysis of the relative changes between H and N showed that H tended to have a greater effect on Ln rMSSD_30s_ after Sub than Supra (*P* = 0.07).

#### Relationships between HR-derived indices, blood gases, and pH

The correlations between post-exercise HR-derived indices and blood variables are presented in Tables 2 and 3. There were small-to-moderate correlations between vagal-related HRV indices and PaO_2_ following Sub. Following Supra, small-to-very large correlations were noted between vagal-related HRV indices and blood variables. The stepwise multiple regression analysis showed that none of the selected blood measures could determine LnHF values following Sub. In contrast, pH (*P* < 0.001, *r*^2^ = 0.20; moderate relationship; *P* = 0.001) was a significant determinant of LnHF following Supra. When all data was pooled (i.e., Sub and Supra). pH (*P* < 0.001) and PCO_2_ (*P* = 0.002) accounted for the greater proportion of total variance (*r*^2^ = 0.51; very large relationship; *P* < 0.001).

**Table 2:** **Correlations between post-exercise HRR and HRV indices and blood variables following submaximal (Sub) and supramaximal (Supra) exercise**.

	pH	PaCO_2_	PaO_2_
**SUB**			
HRR_60s_	0.13 (−0.29; 0.50)	−0.22 (−0.55; 0.17)	0.19 (−0.23; 0.55)
HRRτ	−0.17 (−0.53; 0.25)	0.19 (−0.23; 0.55)	0.14 (−0.28; 0.51)
Ln rMSSD	0.02 (−0.17; 0.21)	0 (−0.19; 0.19)	0.29* (0.11; 0.46)
LnHF	0.12 (−0.07; 0.30)	−0.05 (−0.24; 0.14)	0.31* (0.13; 0.47)
Ln LF/HF	−0.10 (−0.28; 0.09)	0.12 (−0.07; 0.30)	−0.30* (−0.46; −0.12)
**SUPRA**			
HRR_60s_	0.65* (0.34; 0.83)	−0.16 (−0.53; 0.26)	0.10 (−0.31; 0.48)
HRRτ	−0.84* (−0.93; −0.66)	0.24 (−0.18; 0.58)	−0.12 (0.50; 0.30)
Ln rMSSD	0.41* (0.24; 0.56)	−0.11 (−0.29; 0.08)	0.03 (−0.16; 0.22)
LnHF	0.46* (0.30; 0.60)	−0.25* (−0.42; −0.06)	0.07 (−0.12; 0.26)
Ln LF/HF	−0.07 (−0.26; 0.12)	0.22 (0.03; 0.39)	−0.13 (−0.31; 0.06)

Pearson’s *r* (90% confidence interval) for absolute difference between the final heart rate at exercise end and the heart rate recorded 60 s later (HRR_60s_), time constant decay of heart rate recovery (HRRτ), natural logarithm of the squares root of the mean of the sum of the squares of differences between adjacent normal *R*–*R* intervals (Ln rMSSD), natural logarithm of the power spectral density in high frequency (LnHF), natural logarithm of the sympathovagal balance index (Ln LF/HF), blood acidosis (pH), carbon dioxide partial blood pressure (PaCO_2_), O_2_ partial blood pressure (PaO_2_). *: Significant correlation with *P* < 0.05.

**Table 3:** **Determinants of post-exercise parasympathetic reactivation, as inferred from a vagal-related heart rate variability index (i.e., LnHF)**.

Variable	Coefficient	*P*	*r*^2^ Regression *P*	Pearson’s *r*
**SUB**				
Intercept	−30.33		*r*^2^ = 0.08	*r* = 0.29	
PaO_2_ (mmHg)	0.02	0.012	*P* = 0.07	(0.08; 0.48)
PaCO_2_ (mmHg)	0.01	0.43	
Ph	4.50	0.40	
**SUPRA**				
Intercept	−61.73		*r*^2^ = 0.20	*r* = 0.44	
PaO_2_ (mmHg)	0.003	0.75	*P* = 0.001	(0.24; 0.60)
PaCO_2_ (mmHg)	−0.007	0.77	
pH	9.18	<0.001	
**POOLED**				
Intercept	−73.69		*r*^2^ = 0.51	*r* = 0.72	
PaO_2_ (mmHg)	0.01	0.051	*P* < 0.001	0.64; 0.79)
PaCO_2_ (mmHg)	0.01	0.002	
pH	10.52	<0.001	

*Stepwise multiple regression analysis. Coefficient of determination (*r*^2^) illustrating the relationships between post-exercise HRV in the high frequency band (LnHF; natural logarithm of the spectral power density in high frequency band) blood O_2_ partial pressure (PaO_2_), blood carbon dioxide partial pressure (PaCO_2_), and blood acidosis (pH). Pearson’s *r* (90% confidence intervals) were also calculated. Sub: submaximal exercise, Supra: supramaximal exercise, and Pooled: submaximal and supramaximal exercises together*.

## Discussion

To the best of our knowledge, the effect of normobaric hypoxia on post-exercise parasympathetic reactivation had never been examined. Partially in accordance with our hypothesis, vagal-related HRV indices were delayed in hypoxic conditions, but only following submaximal exercise. The effect of hypoxia was not apparent following supramaximal exercise.

Both SpO_2_ and PaO_2_ were lower in hypoxia, while there was no between environmental-condition differences in PaCO_2_ and pH (Figures 2 and 3). The fact that hypoxia induced similar changes in these two latter blood variables following both Sub and Supra (Figures 2 and 3) was crucial for the present study design; the respective influence of blood O_2_ and pH content on post-exercise HR-derived indices could be accurately compared after both exercise intensities. At rest, we found no significant effect of hypoxia on either HR or HRV indices. These results are in line with the belief that altitude should generally exceed 3500 m to substantially alter cardiac parasympathetic activity at rest (Yamamoto et al., 1996; Buchheit et al., 2004), and suggest that the hypoxia-induced changes in post-exercise parasympathetic reactivation observed in the present study were unlikely affected by changes in resting autonomic activity. Since direct measurement of vagal nerve activity was not possible in this study, we used HRR and HRV to non-invasively assess parasympathetic reactivation (Buchheit et al., 2007b). Hypoxia can increase sympathetic activation during exercise (Yamamoto et al., 1996), which can directly affect post-exercise parasympathetic reactivation. Therefore, to ensure a similar level of cardiac sympathetic activation during submaximal exercise in both conditions, exercise HR was matched while adjusting running speed if needed. This was essential to assess parasympathetic reactivation without the possible confounding effect of differences in relative exercise intensity (and associated differences in sympathetic activity, i.e., central command). Finally, sympathetic activation is modulated by the degree of hyperventilation during hypoxia, possibly through stimulation of pulmonary afferents (Somers et al., 1989b). While this could have differently affected parasympathetic reactivation in hypoxia and normoxia, there was no difference in breathing frequency between both experimental conditions (as inferred from HF_peak_ data, Table 1). Therefore, respiratory patterns were unlikely to affect the present results (Table 1).

### Effect of hypoxia on parasympathetic reactivation following submaximal exercise

Following submaximal exercise, hypoxia substantially reduced HRR_60s_ compared with normoxia (Table 1). This is consistent with our first hypothesis, i.e., that low PaO_2_ level may delay post-exercise HRR (Ba et al., 2009; Mahe et al., 2011). There was however no clear correlation between HRR_60s_ and PaO_2_ level (Table 2). The possible relationship between PaO_2_ and HRR_60s_ is likely population-dependent, with correlations evident in unhealthy individuals (who experience very low PaO_2_ levels), but not for healthy individuals (Ba et al., 2009; Mahe et al., 2011; Table 2). For instance, Ba et al. (2009) reported a reduced HRR in patients presenting high blood hypoxemia, but not in healthy subjects. In contrast to HRR_60s_, HRRτ was not different in hypoxia compared with normoxia (Table 1). The discrepancies in the responses of HRR_60s_ and HRRτ following submaximal exercise in hypoxia may be related to the fact that these variables span different time frames, to their different mathematical entities or to their different underlying mechanisms (Buchheit et al., 2007b). When HRR_60s_ was expressed as a percentage of the overall HR recovery amplitude (%HRR_60s_), there was no difference anymore following Sub, in agreement with HRRτ results. Therefore, it is difficult to decipher whether the lowered HRR_60s_ observed in hypoxia is effectively related to a slower parasympathetic reactivation, or more to an increased resting HR during the end-recovery period. While further investigations are therefore warranted to better understand the responses of these two variables (HRR_60s_ and HRRτ) to hypoxia, the following paragraph on HRV measures still highlights an autonomic effect of hypoxia.

Vagal-related HRV indices following Sub were also depressed in hypoxia (Table 1, Figure 5). These results are in agreement with previous findings, where severe hypoxia (i.e., simulated altitude exceeding 3500 m) substantially decreased vagal activity at rest and during exercise (Yamamoto et al., 1996; Buchheit et al., 2004). The present findings showed for the first time that moderate hypoxia (simulated altitude of 2400 m) is also likely to delay parasympathetic reactivation following submaximal exercise. Despite controversies (Eckberg, 1997) and its low reliability (Al Haddad et al., 2011), the LF/HF ratio is generally used as an index of the sympathovagal balance. The greater Ln LF/HF observed in H suggests a shift toward a sympathetic predominance after exercise in hypoxia (Table 1). This accentuated sympathetic activity was unlikely related to changes in central command (since exercise HR was matched, and running speed tended to be lower, ES = 0.37), but rather related to peripheral chemoreflex activation as a consequence of a lower PaO_2_ concentration. This is further supported by the small-to-moderate correlations reported between the different vagal-related HRV indices and PaO_2_ concentration (Table 2). Chemoreflex activation is indeed a major determinant of parasympathetic reactivation (Rowell et al., 1989; Halliwill et al., 2003, Kara et al., 2003), at least following submaximal exercise.

### Effect of hypoxia on parasympathetic reactivation following supramaximal exercise

Following supramaximal exercise, there was no difference in post-exercise HRR and HRV indices between hypoxia and normoxia (Table 1, Figure 5). While this can be seen as paradoxical with respect to observations from previous studies (Ba et al., 2009; Mahe et al., 2011), care should be taken when comparing different populations (unhealthy vs. healthy) and the nature/cause (physiological hypoxemia vs. simulated hypoxic exposure) of blood hypoxemia. Differences in exercise intensity (supramaximal vs. maximal or submaximal) can also explain these discrepancies. The supramaximal exercise used in the present study (i.e., all-out 20-s sprint) was likely to elicit a greater anaerobic glycolytic participation than a constant submaximal (present Sub data and those from Mahe et al. (2011)) and/or incremental exercise (eliciting 90% of subjects’ maximal HR; Ba et al., 2009). Compared with these latter lower-intensity exercises, the greater metabolite accumulation in the blood observed after Supra was likely associated with an almost-maximal autonomic activity perturbation (Buchheit et al., 2007a); the superimposed hypoxic stimulation was therefore unlikely to further impair cardiac autonomic activity (Figure 4). This suggests that, in the context of an already heightened sympathetic activation (i.e., evidenced by the maximal effort and a low post-exercise pH value), an additional stimulation of the chemoreflex activity via decreased PaO_2_ is unlikely to impair parasympathetic reactivation to a greater extent. The fact that the low O_2_ pressure (FiO_2_ = 15.4%) did not affect post-exercise parasympathetic reactivation suggests that high blood acidosis overwhelmed the possible effect of the reduction in PaO_2_ (Table 2). Multiple regression analyses (Table 3) supported the fact that system-stress metabolite accumulation in the blood is likely the major determinant of parasympathetic activity restoration following exercise (Buchheit et al., 2007a; Seiler et al., 2007; Buchheit et al., 2008); as blood pH was the variable accounting for the greatest proportion of variance. This is in agreement with results from a previous study, where it was showed that the baroreflex-mediated increase in parasympathetic activity (lying supine vs. standing) was abolished under heightened sympathetic stimulation (Buchheit et al., 2009). Although speculative, present and previous (Buchheit et al., 2009) findings suggest that peripheral chemoreceptors located in carotid and aortic bodies (activated by a fall in O_2_; Kara et al., 2003) have a lower influence on ANS under high acidosis condition than central chemoreceptors (which are supposed to primarily respond to the fall in blood pH and hypercapnia).

## Conclusion

Following submaximal running exercise, post-exercise parasympathetic reactivation was impaired in normobaric hypoxia (FiO_2_ = 15.4%) compared with normoxia. However, the effect of hypoxia on post-exercise cardiac ANS function was not apparent when the exercise was supramaximal. This suggests that metaboreflex and central chemoreflex activation via blood metabolite accumulation (i.e., which induce a low blood pH) is likely the stronger determinant of parasympathetic activity restoration following exercise. Further studies are required to assess the effect of the severity of hypoxia on post-exercise parasympathetic reactivation and to assess post-exercise blood pressure variability to monitor sympathetic activity during the recovery period (Buchheit et al., 2011). Moreover, future studies should also be conducted with the use of hyperoxic gas mixtures during the recovery period to better define the importance of O_2_ availability on post-exercise parasympathetic reactivation.

### Limitations

In this study, blood samples were drawn from the finger, and therefore changes in the PaO_2_ and PaCO_2_ concentration could present limitations compared with radial artery sampling (Sauty et al., 1996). While breathing frequency (as inferred from HF_peak_ data) was likely similar between the two experimental conditions in the present, tidal volume, which can influence vagal-related indices (Hirsch and Bishop, 1981), was not controlled in our study. Examining parasympathetic reactivation under controlled vs. uncontrolled breathing patterns (accurately monitored via direct measures and not estimated via HF_peak_) would therefore help clarifying the exact influence of ventilation on the observed changes in post-exercise parasympathetic activity. In the present study also, we did not measure blood pressure dynamics. The effect of local vasodilatation, induced by hypoxia, on post-exercise HR recovery could not be examined and need further investigation. The lack of a randomization between exercises (i.e., Sub always before Supra) is another limitation of the present study. A possible order effect was however unlikely to affect present results. Since Sub was associated with a limited level of glycolytic anaerobic energy release (Figure 2), this latter exercise was unlikely to affect the HR responses to Supra. Finally, we have deliberately recruited moderately trained subjects because we expected that they would be able to handle the demands of the present experiments. Trained subjects often display faster parasympathetic reactivation (Buchheit and Gindre, 2006), so the present results should be viewed with caution when inferences are being made into the effect that these interventions may have in different population. Whether individuals with low physical activity and/or parasympathetic levels show a similar response to hypoxia requires further investigation.

## Conflict of Interest Statement

The authors declare that the research was conducted in the absence of any commercial or financial relationships that could be construed as a potential conflict of interest.
